# Age at Diagnosis and Baseline Myelomalacia Sign Predict Functional Outcome After Spinal Meningioma Surgery

**DOI:** 10.3389/fsurg.2021.682930

**Published:** 2021-07-02

**Authors:** Johannes Wach, Mohammed Banat, Patrick Schuss, Erdem Güresir, Hartmut Vatter, Jasmin Scorzin

**Affiliations:** Department of Neurosurgery, University Hospital Bonn, Bonn, Germany

**Keywords:** functional outcome, surgery, recurrence, spinal meningioma, surgical adverse events

## Abstract

**Objective:** Spinal meningioma (SM) accounts for 12% of all meningiomas. Clinical and immunohistochemical factors were analyzed with regard to functional outcome, surgical adverse events, and tumor recurrence.

**Methods:** One-hundred and twenty-three consecutive SM patients underwent surgery and were retrospectively reviewed with regard to demographic parameters, imaging features, neurological function, and immunohistochemical items. Neurological function was graded according to the Modified McCormick Scale (MMS) and dichotomized as “good (grade I + II)” and “poor (grade III–V)” function.

**Results:** One-hundred and fourteen (92.7%) WHO grade I and 9 (7.3%) WHO grade II SM were included in this study. Univariate analysis identified a baseline T2 hyperintensity of the spinal cord, baseline symptom duration ≥4 weeks, age ≥66 years, and dural tail sign as predictors of poor MMS. Baseline T2 hyperintensity of the spinal cord [Odds ratio (OR) = 13.3, 95% CI = 3.4–52.1, *p* < 0.001] and age ≥66 years (OR = 10.3, 95% CI = 2.6–41.1, *p* = 0.001) were independent predictors of a poor MMS grade at discharge after SM surgery in the multivariate binary logistic regression analysis. The 12- and 24-month recurrence-free survival rates were 98.7 % (1/80) and 94.7% (2/38), respectively. In those patients with tumor recurrence of the SM, highly increased MIB-1 (≥5%) labeling indices were observed.

**Conclusion:** Baseline T2 hyperintensity, especially in the elderly patients, is a strong predictor of poorer recovery after spinal meningioma surgery. SMs with high proliferative activity should be followed-up closely despite maximal safe resection.

## Introduction

Spinal meningioma (SM) constitutes 25–46% of all primary spinal tumors, while it represents only 12% of all meningiomas (1–5). In fact, 90% of SMs are intradural, 5% are extradural, and 5% are intra- and extradural. There is a female predominance with SMs, and the sex ratio is 4:1 (female: male) (6, 7).

The typical clinical presentation at onset consists of pain followed by motor deficits, gait instability, and sphincter dysfunction (8). A milestone in the surgical management of spinal tumors was the introduction of intraoperative MEP and SEP monitoring to preserve neurological functions (9). Furthermore, it is suggested that anterior location, prolonged duration of baseline symptoms before surgery, WHO grade >I, and Simpson grade II and III resections are predictive factors of a poor functional outcome (10).

SMs are predominantly benign tumors and the treatment of choice is primarily the gross total resection (Simpson grade I–III) of the lesion. The reported rates of recurrence vary largely between 1.3 and 13% (4, 7, 8, 10–15). Due to the known potential good oncological long-term outcome surgery is the only appropriate treatment for tumor control in this predominantly benign tumor entity. It is important to achieve a good neurological functional outcome after surgical resection to maintain the ambulatory ability and quality of life. Therefore, it is of paramount importance to know potential predictive factors of functional outcome after surgery for sporadic benign SMs.

The scope of this study is to investigate our series of sporadic benign spinal meningiomas below the craniocervical junction with regard to functional outcome, surgical adverse events, and rates of tumor recurrence after SM surgery and to elucidate the predictive factors.

## Methods

### Patient Population

This study reviews 123 consecutive patients with SM who had undergone surgical resection between 2000 and 2019. The scope of this study is focused on the analysis of SMs located below the craniocervical junction. Patients with anterior foramen magnum meningiomas, craniocervical meningiomas (occipital bone, C1, C2), a recurrent meningioma after radiotherapy, and patients with a neurofibromatosis type 2 were excluded due to the differences regarding clinical signs, histopathology, and therapy (16–19).

### Data Recording and Neurological and Neuroradiological Definitions

Clinical information including age, sex, comorbidities, Karnofsky Performance Status, body mass index (BMI), and the American society of anesthesiologists physical status classification system (ASA) were collected and entered into a computerized database (SPSS, version 27 for Mac, IBM Crop., Armonk, NY). Neurological examination was performed preoperatively, postoperatively at discharge, and at 3- and 12-months follow-up visits by institutional neurosurgeons. Overall neurological function, especially the ambulatory ability, was evaluated using the modified McCormick Scale (MMS) (20). MMS was dichotomized and defined as “good (I + II)” and “poor (III–V)” function (21). Duration of preoperative symptomatic was dichotomized into short (<4 weeks) and long (≥4 weeks) (22). Magnetic resonance imaging (MRI) was performed within 72 h before surgery, as well as 3 and 12 months postoperatively. Further follow-up MR images were performed on an annual basis (23). Radiological tumor recurrence was defined as a visible tumor progression on a follow-up MR image at least 1 year after surgery (24). Myelomalacia was defined as patients presenting with high intensity signal changes on T2-weighted images (25). MR-images were interpreted by independent neuroradiologists.

### Surgical Procedure

Indications for surgery included local back pain without the presence of competing spinal pathologies, neurological dysfunctions, and spinal cord compression. Surgical management was dependent on the site of dural attachment of the tumor, tumor size, as well as the spinal level of the meningioma. Hemilaminectomy or laminoplasty was performed to preserve functional stability of the spine. There were no inclusion or exclusion criteria with regard to the decision to use either hemilaminectomy or laminoplasty. Resection of the dentate ligament became necessary if the tumor had a ventral dural attachment. Closure of the dura was performed with continuous silk sutures and additional sealed with TachoSil® (Fibrin Sealant Patch).

### Surgical Adverse Events

Postoperatively, adverse events, such as wound healing disorders, deep and superficial surgical site infections SSIs and CSF fistulas requiring revision surgeries were recorded. SSIs were identified based on postoperative clinical notes, using the Centre for Disease Control definition of infection (infection involving superficial incisional, deep incisional, and/or organ/space SSI) (26).

### Histopathology

Paraffin sections were stained with hematoxylin-eosin, and neoplastic areas were delineated. Histopathological grading was performed according to the 2016 WHO criteria. The classification and grading of meningiomas did not undergo further revisions in 2016 (27). Immunohistochemistry was performed with Anti-Ki67 (Clone Ki-67P, dilution 1:1,000, Dianova), anti-CD45 (Clone 2B11 + PD7/26, dilution 1:1,000, DAKO), and anti-CD68 (Clone KP1, dilution 1:1,000, DAKO) Afterwards, the entire section was systematically examined using an optical grid on high-power fields for the presence of immunoreactivity. The area of densest staining was searched in every slide, and counting was performed in this area to determine the Molecular Immunology Borstel (MIB)-1 labeling index. The MIB-1 labeling index was dichotomized and defined as “low proliferative” (<5%) and “high proliferative” (≥5%) according to the created receiver operating characteristic (ROC) curve.

### Statistics

Statistical analysis was performed using the IBM SPSS statistics for windows version 25 (Armonk, NY: IBM Corp). Parametric mean values of the variables were analyzed by the Student's *t*-test and categorical variables by the two-sided Pearson's chi-squared test. Univariate analysis was performed to determine the effect of clinical, histopathological, and neuroimaging variables on functional outcome. Afterwards, multivariate binary logistic regression analysis was performed to find independent predictors of functional outcome. Correlations between quantitative and continuous variables were investigated by the Pearson's correlation coefficient. ROC analysis was performed to investigate the cut-off value for the MIB-1 labeling index. Dependencies of the variables were analyzed in a multivariate binary logistic regression analysis model. Variables with significant probability values on univariate analysis were considered as potentially independent on multivariate analysis. Recurrence-free survival was analyzed using the Kaplan-Meier method (log-rank test). Cox regression analysis was used to find predictive factors for the variable of recurrence in the univariate analysis. A time dependent multivariate Cox regression with the inclusion criteria of a probability value < 0.05 was performed.

## Results

### Baseline Patient Characteristics

There was a female predominance among the SMs and the mean age was 65.6 (SD: ±12.3) years. Tumors were predominantly located in the thoracic spine (90/123; 73.2%). Obesity [Body mass index (BMI) ≥30.0] was observed in 39 patients (31.7%). The sites of dural attachment of the tumor were distributed homogeneously. Baseline hyperintensity of the spinal cord in the T2-weighted MRI was observed in 52.8% of cases. Calcification of the tumor was observed in 11 patients (8.9%).

### Surgical and Neuropathological Results

Laminoplasty was performed in 7 cases (5.7%). Closed suction drains were placed in 71 patients (57.7%). Median operation length (25th−75th percentile) was 178.9 min (130.0–204.0). Histopathological examination revealed 114 (92.7%) WHO grade I and 9 (7.3%) WHO grade II SMs. Malignant cases were not observed. The median (range) MIB-1 labeling index value was 4.0 (2.0–12.0) in 123 patients. Further clinical and imaging features are summarized in Table 1.

**Table 1:** Baseline clinical, imaging and surgical characteristics of patients (*n* = 123).

**Age (mean ± SD)**	**65.6 (± 12.3)**
Sex	
** Female**	**94 (76.4%)**
** Male**	**29 (23.6%)**
ASA PS	
** 1**	**21 (17.1%)**
** 2**	**69 (56.1%)**
** 3**	**33 (26.8%)**
**Obesity (BMI ≥ 30.0)**	**39 (31.7%)**
**Diabetes mellitus type 2**	**20 (16.3%)**
**Karnofsky Performance Status [median (25th percentile, 75th percentile)]**	**80.0 (70.0, 90.0)**
Preoperative symptoms	
** Pain**	**85 (69.1%)**
** Sensory deficits**	**49 (39.8%)**
** Motor deficits**	**26 (21.1%)**
** Gait ataxia**	**17 (13.8%)**
** Bladder and/or bowel dysfunction**	**5 (4.1%)**
** Incidental**	**4 (3.3%)**
Preoperative modified Mccormick scale	
** I**	**48 (39.0%)**
** II**	**35 (28.5%)**
** III**	**22 (17.9%)**
** IV**	**15 (12.2%)**
** V**	**3 (2.4%)**
Dural attachment (one patient was excluded because of selective arachnoid attachment)	
** Ventral**	**37 (30.3%)**
** Lateral**	**49 (40.2%)**
** Dorsal**	**36 (29.5%)**
Spinal level	
** Cervical**	**31 (25.2%)**
** Thoracic**	**90 (73.2%)**
** Lumbar**	**2 (1.6%)**
Involved spinal segments	
** ≤ 2**	**110 (89.4%)**
** >2**	**13 (10.6%)**
Myelomalacia (T2-weighted MR-image)	
** Present**	**65 (52.8%)**
** Absent**	**58 (47.2%)**
Dural tail sign (Gd-DTPA-enhanced MR-image)	
** Present**	**18 (14.6%)**
** Absent**	**105 (85.4%)**
Calcification	
** Present**	**11 (8.9%)**
** Absent**	**112 (91.1%)**
Cyst	
** Present**	**2 (1.6%)**
** Absent**	**121 (98.4%)**
Extent of resection according to Simpson grading	
** I**	**59 (48.0%)**
** II**	**59 (48.0%)**
** III**	**3 (2.4%)**
** IV**	**2 (1.6%)**


### Functional Outcome

The mean (±SD) preoperative MMS was 2.11 (±1.13). Good MMS grade (I + II) was observed in 83 cases (67.5%), preoperatively. At discharge, the mean (±SD) MMS was 1.98 (±1.12), respectively (*p* = 0.46). Good functional outcome according to the MMS was observed in 91 patients (74.0%) at discharge after SM surgery. Univariate analysis of potential risk factors of poor MMS at discharge was performed. Cut-off value for age in the prediction of postoperative functional outcome was determined using ROC analysis. ROC analysis showed an AUC of 0.77 (95% CI: 0.68–0.86 *p* < 0.001, Youden index = 0.38) to detect poor MMS. The sensitivity and specificity of a cut-off value set at ≥66 years were 74.2 and 64%, respectively.

Univariate analysis revealed age ≥66 years, baseline myelomalacia, duration of baseline symptoms, and dural tail sign as statistically significant in terms of being associated with poor MMS (III–V) (Table 2). Baseline myelomalacia in the T2-weighted MRI was further analyzed with regard to potential associations with age, duration of baseline symptoms, tumor location, dural attachment site, WHO grade, tumor size and involved spinal segments (Table 3). Fisher's exact test (two-sided) revealed that a baseline symptom duration ≥4 weeks (54/91 (59.3%) is significantly associated with baseline myelomalacia in the T2-weighted MR compared to patients with a shorter baseline symptom duration [11/32 (34.4%); *p* = 0.02]. The patients with an age ≥66 years or <66 years were further analyzed with regard to BMI, cigarette abuse (yes/no), presence of malignant tumors, Karnofsky Performance Status, and the American society of anesthesiologists physical status classification system (ASA). The mean (±SD) BMI in patients aged <66 years was 27.71 ± 5.35, whereas patients with an age ≥66 years had a mean (± SD) BMI at 28.87 ± 5.13 (independent *t*-test: *p* = 0.30). Ten (10/48; 20.8%) patients with an age <66 years were smokers, and 10 (10/75; 13.3%) of the patients with an age ≥66 years were smokers, respectively [Pearson's chi-squared test (two-sided): *p* = 0.27]. Malignant tumors were found in 3 (3/48; 6.3%) patients in the group of patients aged <66 years, whereas 7 (7/75; 9.3%) patients in the age ≥66 years group had a malignant tumor disease [Pearson's chi-squared test (two-sided): *p* = 0.54]. A baseline Karnofsky Performance Status <80 was observed in 13 (13/48; 27.1%) patients aged <66 years, and in 24 (24/75; 32.0%) patients of the age ≥66 years group, respectively [Pearson's chi-squared test (two-sided): *p* = 0.56]. Ten (10/48, 20.8%), 29 (29/48; 60.4%), and 9 (9/48; 18.8%) had an ASA class of 1, 2, and 3 in the group of patients with an age <66 years, whereas 11 (11/75; 14.7%), 40 (40/75; 53.3%), and 24 (24/75; 32.0%) had an ASA class of 1, 2, and 3 in the group of patients aged ≥66 years [Fisher's exact test (two-sided): *p* = 0.24].

**Table 2:** Clinical and imaging features associated with the modified Mccormick scale.

**Variable**	**Preoperative “good MMS” (*n =* 123)**	***p*-value**	**Postoperative “good MMS” (*n* = 122)**	***p*-value**
**Age**				
≥66 years	39/66 (59.1%)	0.03	40/65 (61.5%)	<0.001
<66 years	44/57 (77.2%)		51/57 (89.5%)	
**Baseline symptom duration**				
<4 weeks	26/32 (81.3%)	0.053	29/31 (93.5%)	0.005
≥4 weeks	57/91 (62.6%)		62/91(68.1%)	
**Myelomalacia**				
Present	32/65 (49.2%)	<0.001	36/64 (56.3%)	<0.001
Absent	51/58 (87.9%)		55/58 (94.8%)	
**Dural tail sign**				
Present	9/18 (50.0%)	0.09	9/18 (50.0%)	0.02
Absent	74/105 (70.5%)		82/104 (78.8%)	
**Involved spinal segments**				
≤2	76/110 (69.1%)	0.06	83/109 (76.1%)	0.11
>2	5/13 (38.5%)		7/13 (53.8%)	
**Dural attachment (one patient was excluded because of selective arachnoid attachment)**				
Ventral	27/37 (73.0%)	0.53	26/37 (70.3%)	0.49
Dorsal and lateral	56/85 (65.9%)		65/84 (77.4%)	
**Simpson grade**				
I + II	82/118 (69.5%)	0.33	89/117 (76.1%)	0.10
III + IV	2/5 (40.0%)		2/5 (40.0%)	
**WHO grade**				
I	77/114 (67.5%)	0.99	84/113 (74.3%)	0.99
II	6/9 (66.7%)		7/9 (77.8%)	
**MIB-1 labeling index (available in 122 patients)**				
<5%	54/72 (75.0%)	0.90	52/72 (72.2%)	0.40
≥5%	32/50 (64.0%)		37/50 (74.0%)	

**Table 3:** Association between baseline myelomalacia and clinical, imaging and histopathological features [Fisher's exact test (two-sided)].

**Variable**	**Baseline myelomalacia**	**No baseline myelomalacia**	***p*-value**
**Age**			
≥66 years	44 (57.3%)	32 (42.7%)	0.27
<66 years	22 (45.8%)	26 (54.2%)	
**Baseline symptom duration**			
<4 weeks	11 (34.4%)	21 (65.6%)	0.02
≥4 weeks	54 (59.3%)	37 (40.7%)	
**Location**			
Cervical	1 (50.0%)	1 (50.0%)	0.99
Thoracic	48 (53.3%)	42 (46.7%)	
Lumbar	16 (51.6%)	15 (48.4%)	
**WHO grade**			
I	61 (53.5%)	53 (46.5%)	0.73
II	4 (44.4%)	5 (55.6%)	
**Dural attachment site**			
Ventral	22 (59.5%)	15 (40.5%)	0.37
Lateral	22 (44.9%)	27 (55.1%)	
Dorsal	20 (55.6%)	16 (44.4%)	
**Involved spinal segments**			
≤ 2 segments	57 (51.8%)	53 (48.2%)	0.11
>2 segments	8 (80.0%)	2 (20.0%)	

Therefore, multivariate binary logistic regression analysis of predictors potentially associated with poor MMS at discharge was performed. The multivariate analysis showed that the presence of a baseline myelomalacia (OR = 13.30, 95% CI = 3.4–52.1, *p* < 0.001) in the T2-weighted MRI and an age of ≥66 years (OR = 10.30, 95% CI = 2.6–41.1, *p* = 0.001) at time of diagnosis were the only independent significant predictors (Table 4) for poor MMS at discharge. We further investigated the impact of age on the change of the modified McCormick scale between preoperative and postoperative functioning. One patient (1/48; 2.1%) aged <66 years deteriorated with regard to MMS scale at discharge compared to the baseline status, whereas 11 patients (11/75; 14.7%) aged ≥66 years had a poorer MMS at discharge compared to the baseline functioning, respectively (Fisher's exact test: *p* = 0.027).

**Table 4:** Multivariate binary logistic regression analysis of predictors of poor MMS at discharge.

**Characteristic**	**Odds ratio**	**95% CI**	***p*-value**
Age ≥66 years	10.30	2.6–41.1	0.001
Baseline symptom duration ≥4 weeks	5.40	1.0–29.40	0.053
Dural tail sign	1.90	0.5–7.0	0.35
Baseline myelomalacia	13.30	3.4–52.1	<0.001

**Figure 1:**
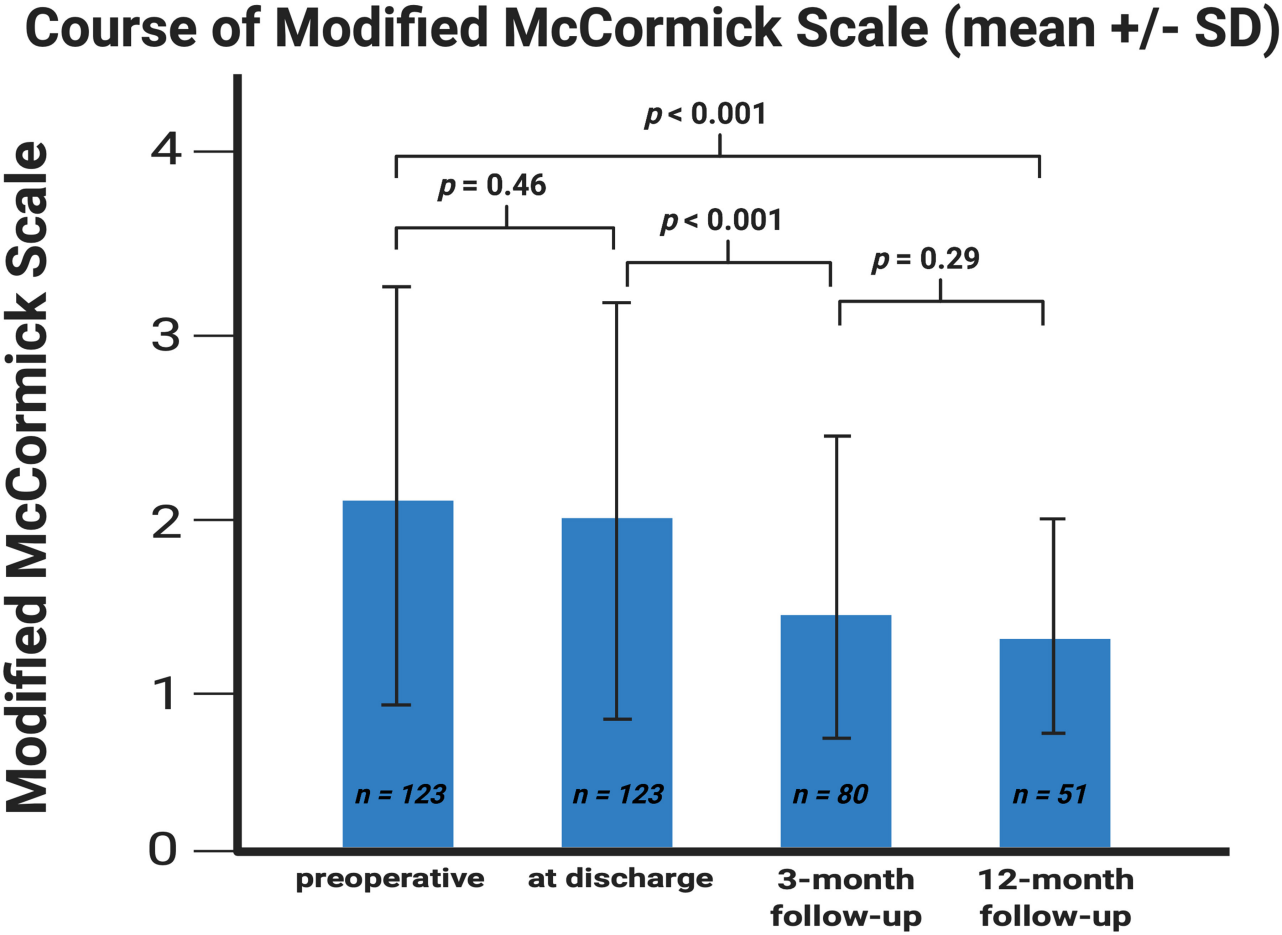
Error bar showing the means and standard deviation of the Modified McCormick scale. X-axis represents the baseline at discharge and at the 3- and 12-month follow-up examinations. Y-axis represents the Modified McCormick scale. *P*-values of the paired *t*-test comparing the mean values are shown. The means (±SD) were 2.11 ± 1.13, 1.98 ± 1.13, 1.54 ± 0.84, and 1.40 ± 0.63 (sorted in chronological ascending order).

In 39 (77.2%) of 51 patients, in whom postoperative follow-up was available until 12 months after surgery, the neurological function graded according to MMS improved significantly at 12-months after surgery. Preoperatively, the mean (±SD) MMS was 2.11 (±1.13), whereas after 12 months the mean dropped to 1.40 (±0.63), respectively (*p* < 0.001). The most significant improvement of the neurological function graded according to MMS was observed during the period between patient discharge and the 3 months follow-up examination (*p* < 0.001) (Figure 1).

### Surgical Adverse Events

Eight (6.5%) surgical adverse events were found in the entire cohort. Surgical site infection was found in one patient (0.8%). Second-look surgery was performed in one case because of an epidural abscess (0.8%) caused by *Staphylococcus aureus* and in two cases due to symptomatic postoperative spinal epidural haematoma (1.6%). Five patients had CSF fistulas following SM surgery requiring secondary interventions (4.1%). Furthermore, 3 patients (2.4%) had postoperative CSF fistulas, which were treated by a lumbar drainage without a revision surgery. Four (4/59; 6.8%) CSF fistulas occurred in the Simpson grade I resection group and one (1/59; 1.7%) CSF fistula was observed in the Simpson grade II resection group, respectively (Pearson's chi squared test (two-sided: *p* = 0.36). Pearson's chi squared test revealed obesity (BMI ≥30) [4/39 (10.3%); *p* = 0.035)] and ventral dural attachment [4/38 (10.5%); *p* = 0.031] as significantly associated with postoperative CSF fistulas. Association between tumor size with regard to the number of involved spinal segments and the prevalence of postoperative CSF fistulas following surgery for SM was analyzed. Five CSF fistulas were observed in the group of SMs involving ≤ 2 spinal segments (5/110) and no CSF fistula was found in the group of SMs involving >2 spinal segments (0/10), respectively [Fisher's exact test (two-sided): *p* = 0.99]. Multivariate binary logistic regression analysis with consideration of obesity (BMI ≥30.0/ <30.0), dural attachment site (ventral/lateral and dorsal), duration of surgery (≥160/ <160 min), and spinal level (cervical/thoracic and lumbar) was performed to analyze potential predictive factors of CSF fistulas. Ventral dural attachment was the only significant predictor of CSF fistulas in the multivariate binary logistic regression analysis (OR: 10.8, 95% CI = 1.0–112.5, *p* = 0.047) (Table 5).

**Table 5:** Multivariate binary logistic regression analysis of predictors of CSF fistula after surgery for spinal meningioma.

**Characteristic**	**Odds ratio**	**95% CI**	***p*-value**
Ventral dural attachment	10.8	1.0–112.5	0.047
Obesity (BMI ≥30.0)	9.6	0.9–98.6	0.058
Duration of surgery (≥160.0 min)	1.2	0.2–9.7	0.84
Cervical location	3.2	0.3–38.0	0.37

### Tumor Recurrence

The mean (range) MRI follow-up time was 28.80 (3.0–120.0) months in 80 (65.0%) patients. The 12- and 24-month recurrence-free survival rates were 98.7 % (1/80) and 94.7% (2/38), respectively.

The first patient was a 65-year-old man presenting with a tumor recurrence at the 12-months follow-up examination. The primary diagnosis was a WHO grade II SM which was completely resected as Simpson grade II. The MIB-1 labeling index was 10%.

The second patient was a 51-year-old man who underwent Simpson grade III resection of a WHO grade I SM with a MIB-1 labeling index of 5%. Simpson grade III resection was performed because the tumor was found to arise from the arachnoid layer, while the spinal dura mater was not involved with the meningioma. Recurrence occurred at the 24-months follow-up examination.

**Figure 2:**
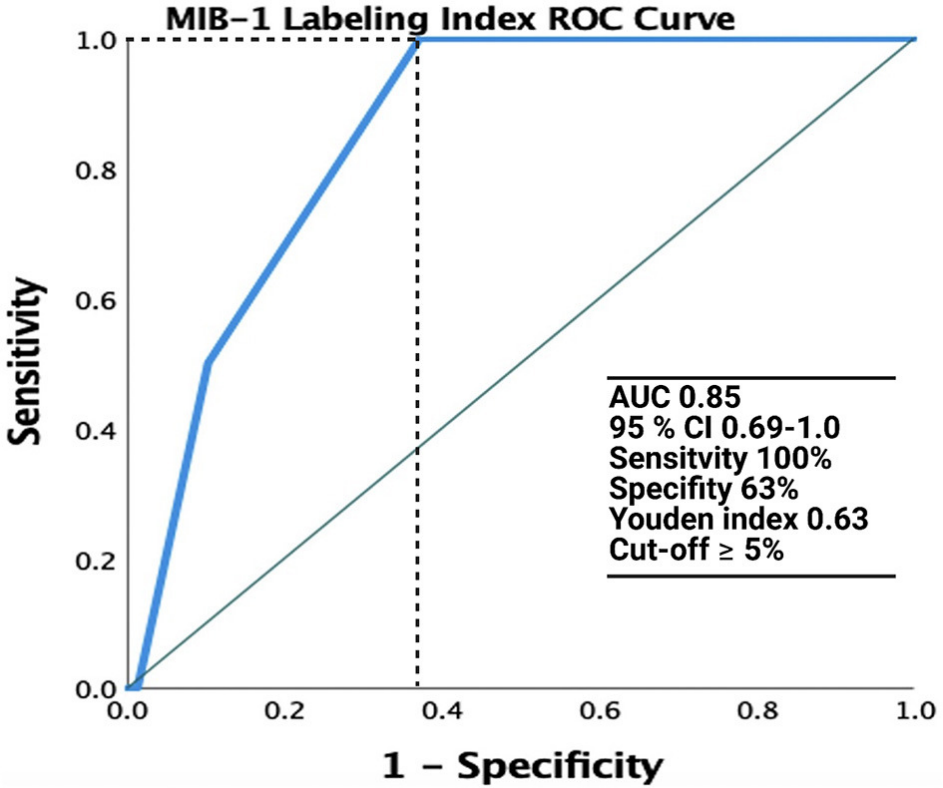
Receiver operating characteristic (ROC) curve illustrating the performance of the MIB-1 labeling index in the prediction of recurrence of spinal meningiomas. The green line represents the reference and the MIB-1 labeling index is shown at the blue line. AUC was 0.85 (95% CI: 0.69–1.0).

The median (range) MIB-1 labeling index value was 4.0 (2.0–12.0) in 123 patients. ROC analysis was performed, showing an AUC of 0.85 (95% CI: 0.69–1.0, *p* = 0.09, Youden index = 0.63) to detect recurrence (Figure 2). The sensitivity and specificity of a cut-off value set at ≥4.5 were 100 and 63%, respectively.

## Discussion

By 1938, Cushing and Eisenhardt had already reported SMs as lesions which should be surgically treated, and they described an oncologically favorable long-term outcome (28). The present study revealed that SM surgery is a safe procedure that results in significant improvement of the postoperative neurological functioning status according to the modified McCormick Scale after surgery in the entire cohort. Baseline signs of myelomalacia in the T2-weighted MRI and an age ≥66 years were associated with poor recovery of neurological function after SM surgery. Ventral dural attachment of SM was found to be significantly associated with an increased risk of postoperative CSF fistulas. Furthermore, a significant association between MIB-1 labeling index ≥5% and tumor recurrence was observed. So far, several studies have confirmed that surgery for spinal meningioma is a safe procedure and favorable long-term functional outcome can be achieved (4, 5, 10, 14, 29, 30). However, the present series spans over a 19-year period and investigates the benign WHO grade I and II SMs, all of which were resected using MEP and SEP monitoring-an established tool in modern spinal tumor surgery (9).

### Functional Outcome

In the present study, an age of ≥66 years and baseline sign of myelomalacia on T2-weighted MRI were associated with a deterioration of neurological function preoperatively as well as in the postoperative period. In our multivariate binary logistic regression analysis, these variables proved to be independent and significant predictors of a poor MMS grade. The role of age at diagnosis regarding recovery was previously reported. A cut-off at the age below 60 years was suggested to be an important predictor of neurological recovery (31). Our investigation confirmed that the age at diagnosis is an important predictor of functional outcome. An age ≥66 years was an independent significant predictor in the multivariate analysis of the functional outcome. Furthermore, we found no heterogeneous distribution among the age groups (<66/≥66 years) with regard to comorbidities, nicotine abuse, Karnofsky performance status, and ASA classification. Additionally, patients aged ≥66 years significantly more deteriorated postoperatively with regard to the functional outcome compared to patients <66 years.

#### Review of Predictors of Functional Outcome

Anterior located lesions were also described to be a risk factor for poor neurological recovery after SM surgery (5, 17, 32). Furthermore, the role of the preoperative duration of symptoms before surgery was mentioned in the literature (5, 31). An effect of the baseline symptom duration regarding the neurological outcome following surgery was observed but closely failed (*p* = 0.053) to be an independent variable in the multivariate binary logistic regression if *p*-value threshold is set at < 0.05. However, in our analysis, symptom duration and anterior location were highly correlated to myelomalacia sign on T2-weighted MR-images, which is basically a sign of spinal cord damage. As far as anatomy of the thoracic spinal canal is concerned, it is relatively narrow compared to the cervical and lumbar regions (33). SMs occur predominantly in this area, and myelomalacia as an imaging sign of spinal cord damage is a conclusive result of this given anatomical disadvantage in the thoracic region. The poor functional outcome of those patients in our study having a baseline myelomalacia sign might be explained by the majority having a late stage or advance stage myelomalacia (25). Frati et al. (34) developed an interesting score predicting the neurological outcome in a retrospective series investigating 173 spinal meningioma cases. This study identified anterior location, operating on a recurrent SM, sphincter involvement, and worse functional grade at onset as potential predictors of the functional outcome. The anterior location was not a significant predictor in our series. Furthermore, we have not included recurrent meningiomas in our analysis, respectively. Some studies reported calcification of the tumors as a predictive variable of postoperative deterioration of neurological function (5, 14). However, in our investigation only 11 patients suffered from a calcified SM. Therefore, this morphological item failed to reach this described significant effect in our series. Morandi et al. (35) investigated 30 patients with a SM aged 70 years or older and found improved functional outcome in all cases. They identified a correlation between the preoperative neurological status and the postoperative outcome. Therefore, they concluded that surgical resection should be performed whenever it is feasible from an anesthesiological point of view with regard to the risk profile of the operation. Furthermore, Gilard et al. (36) investigated predictors of postoperative functional outcome in 87 patients who underwent surgery for SM in a retrospective series. They identified the absence of preoperative neurological signs, anterior location of the meningioma and WHO grade II as predictors of postoperative functional deterioration. In the present series we could not identify those predictors as independent predictors of functional outcome following surgery for SM. Intraoperative neuromonitoring is an essential tool in the modern era of neurosurgery to prevent worsening of neurological outcome during surgery. The present study analyzes a large institutional series between 2000 and 2019. However, we have not included intraoperative neuromonitoring data for analysis due to changes in the medical devices, interobserver bias and incomplete neuromonitoring data. Korn et al. (37) described a multimodality approach by using transcranial motor evoked potential, somatosensory evoked potential and EMG monitoring in a retrospective study analyzing 100 patients who underwent surgery for intradural extramedullary spinal cord tumors. In this series by Korn et al. (37) 22 patients had a spinal meningioma. They showed a specificity of 95%, a sensitivity of 82%, with a negative predictive value of 95%, and a positive predictive value of 82% for detecting neurologic injuries during resection.

Overall, SM surgery is an efficient procedure in order to improve the neurological function. There is a continuous improvement of the mean values of the MMS (Figure 1) in the whole postoperative examination period. Strongest improvement of the function was observed, especially between discharge and the 3-month follow-up examination. At this timepoint, most of the patients completed their postoperative physiotherapy program, which might explain this observation. Preoperatively, 67.5% of patients had a good MMS grade, whereas after 3 months 85.0% had a good MMS grade (*p* < 0.001).

### Surgical Adverse Events

#### Surgical Site Infections

Generally, SM surgery was proved to be a safe procedure. The rate of surgical adverse events requiring secondary surgeries is low in our single-center series. Only one SSI (0.8%) was observed in 123 cases. A large study evaluating the incidence of SSI in 1918 spinal procedures revealed a rate of 2.76% (38). Similarly, in our single-center study evaluating surgical complications after surgery for spinal metastases, the rate of SSI was 2.4% (39).

#### Postoperative CSF Leaks

We found CSF leaks requiring secondary interventions in 4.1% (5/123 cases) of all patients in our series. The literature reports various rates of postoperative CSF leaks in extradural spinal tumor surgery, which vary between 2.5 and 10.4% (40, 41). CSF leaks were observed in 3% in our institutional series investigating surgery for spinal metastases, respectively (39). However, Simpson grade I resection in spinal meningiomas is also associated with CSF fistulas following surgery. In cases of SMs with ventral dural attachment site, reconstruction of the dura is very difficult and CSF fistulas still occur despite the use of thoracolumbar fascia or artificial dura (42). In our series 59 patients underwent Simpson grade I resection, and various techniques were used to facilitate duraplasty (e.g., fibrin sealant patches, silk sutures). In order to ensure spinal cord protecting surgery, it is often necessary to choose an extended approach in cases where the spinal meningioma is aligned ventrally in order to limit potential collateral damage from surgical preparation. This widened wound area and exposure of the dura as well as the more difficult dural closure might be used to explain the increased incidence of postoperative CSF fistulas in these complex cases.

### Tumor Recurrence

The rate of tumor recurrence in our series was 2.5% (2/80). This rate is in accordance with those reported in the literature, which range between 1.3 and 13% (4, 7, 8, 10–15). A large series investigating 173 SMs revealed a tumor recurrence rate of 2.3% (10). In the present series, the 12- and 24-month recurrence-free survival rates were 98.7% (1/80) and 94.7% (2/38), respectively. In those patients who had a tumor recurrence of their SM in the present series, highly increased MIB-1 labeling indices were observed. ROC curve analysis revealed a strong diagnostic ability of a MIB-1 labeling index ≥5% in the prediction of meningioma recurrence. In a study by Perry et al. (43), in which 425 patients underwent complete resection of primary meningiomas, a MIB-l labeling index ≥4.2 was suggested to be strongly associated with tumor recurrence in cranial meningiomas. Therefore, the MIB-1 labeling index seems to be also an important predictor of tumor recurrence in patients who underwent a sufficient surgical treatment. However, Roser et al. (44) found in an analysis of 25 cases that the proliferation potential of spinal meningiomas differs significantly from intracranial meningiomas measured by the MIB-1 labeling index. Despite lower proliferation rates of spinal meningiomas in this study, time to recurrence in spinal and cranial meningiomas was comparable. Simpson grade I and II are known positive predictors in terms of tumor recurrence (12, 45). However, a recent meta-analysis of spinal meningiomas found no statistically significant differences between Simpson grade I or II in terms of recurrence (42). Recently, a new technique was presented by Saito et al. (46) which leaves the outer layer of the dura intact and the tumor attachment is resected along with the inner dural layer. This method was predominantly introduced and used for tumors with a ventral dural attachment in order to reduce the rate of CSF fistulas and the authors observed also no recurrence in a retrospective series of 10 patients with a follow-up of 10 years (47). Therefore, this seems to be an alternative approach to Simpson grade I resection with regard to long term tumor control and avoidance of CSF fistulas.

## Limitations of the Study

Limitations of our investigation include the retrospective design and the common drop-out of patients in the clinical follow-up examinations in retrospective oncological studies. Additionally, myelomalacia was not further classified in early, intermediate and late stage according to known MR criteria (23). Follow-up imaging was available in only 80 (65.0%) of the 123 cases and the number of recurrent tumors is very low. Consequently, the results of our analysis of tumor recurrence regarding the rare morphological signs, including calcification or MR-imaging features such as the dural tail sign, have to be interpreted with caution due to the mentioned limitations and interobserver variability in imaging interpretation. Furthermore, the number of events with regard to surgical adverse events and tumor recurrence are low. Therefore, the results of the multivariate analysis with regard to those mentioned endpoints have to be investigated and confirmed a in future larger series. Histopathologically, there is an interobserver variability regarding the determination of the MIB-1 labeling index in our collective because of the long time period which was investigated. Additionally, no determination with a digital image analysis system was performed to determine the MIB-1 labeling index, which might be more objective due to the fact that greater number of microscopic fields can be analyzed with this method (48).

## Conclusion

In summary, surgical resection of SMs is a safe procedure and postoperative improvement of the neurological functional status was evident throughout the present cohort. Patients with baseline myelomalacia sign on T2-weighted MRI and those with an age ≥66 years should be informed about poorer postoperative recovery with regard to the functional outcome after SM surgery.

## Data Availability Statement

The raw data supporting the conclusions of this article will be made available by the authors, without undue reservation.

## Ethics Statement

The studies involving human participants were reviewed and approved by ethics committee of the University Hospital Bonn. Written informed consent for participation was not required for this study in accordance with the national legislation and the institutional requirements.

## Author Contributions

Data acquisition was performed by JW. JW and JS performed the study design, methodology, statistical analysis, and writing and creation of figures. Supervision of the study was done by JS. Proofreading was done by MB, EG, PS, HV, and JS. All authors contributed to the article and approved the submitted version.

## Conflict of Interest

The authors declare that the research was conducted in the absence of any commercial or financial relationships that could be construed as a potential conflict of interest.
