# Supplementary figures and images for: Age at Diagnosis and Baseline Myelomalacia Sign Predict Functional Outcome After Spinal Meningioma Surgery
Source: Front Surg. 2021 Jul 2;8:682930. doi: 10.3389/fsurg.2021.682930 (PMC8282826; doi:10.3389/fsurg.2021.682930)

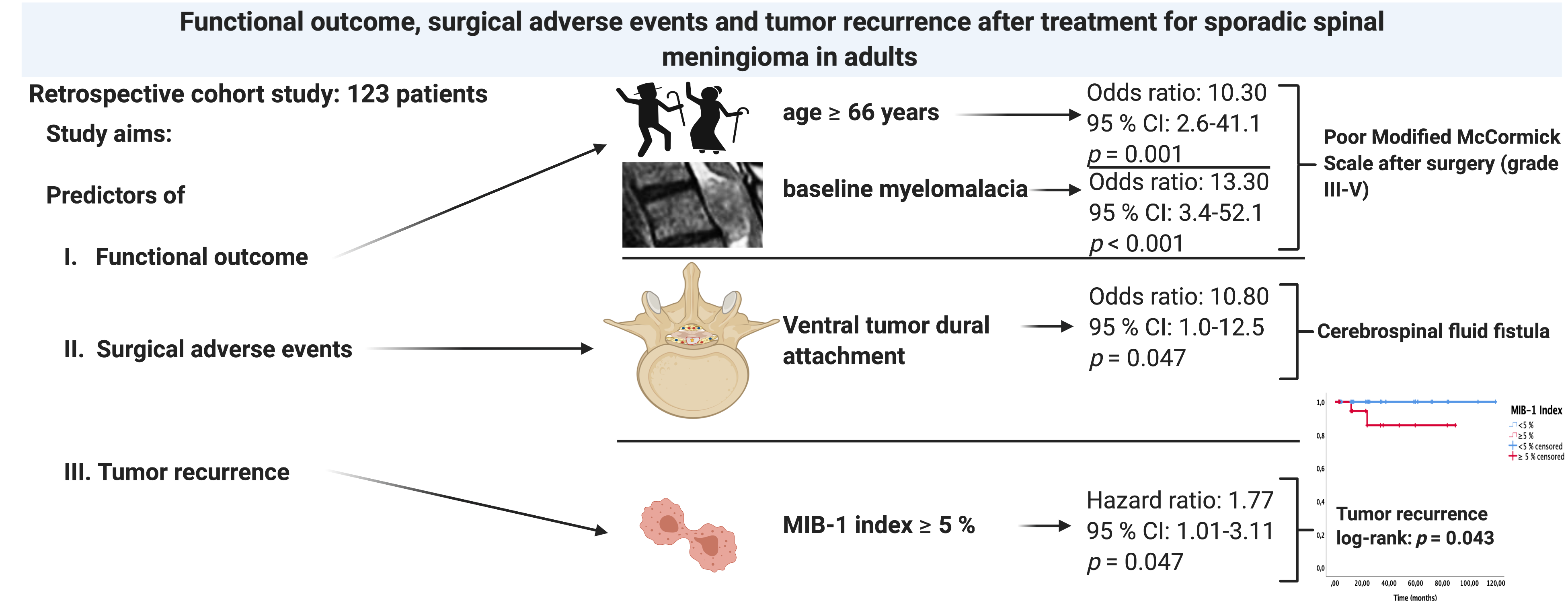

Supplement: Supplementary file 1 [file Image_1.TIFF]
